# Distribution of *Culex pipiens* life stages across urban green and grey spaces in Leiden, The Netherlands

**DOI:** 10.1186/s13071-024-06120-z

**Published:** 2024-01-29

**Authors:** Louie Krol, Melissa Langezaal, Lisa Budidarma, Daan Wassenaar, Emilie A. Didaskalou, Krijn Trimbos, Martha Dellar, Peter M. van Bodegom, Gertjan W. Geerling, Maarten Schrama

**Affiliations:** 1 Institute of Environmental Sciences, Leiden University, Leiden, The Netherlands; 2 Deltares, Daltonlaan 600, Utrecht, The Netherlands; 3 Department of Environmental Science, Radboud Institute for Biological and Environmental Sciences, Radboud University, Nijmegen, The Netherlands

**Keywords:** Community ecology, *Culex pipiens*, Climate resilience, Dispersal, Environmental DNA, Nature-based solutions, Mosquito control

## Abstract

**Background:**

There is an urgent need for cities to become more climate resilient; one of the key strategies is to include more green spaces in the urban environment. Currently, there is a worry that increasing green spaces might increase mosquito nuisance. As such, this study explores a comprehensive understanding of how mosquitoes utilise contrasting grey and green habitats at different life stages and which environmental factors could drive these distributions.

**Methods:**

We used a setup of six paired locations, park (green) vs. residential (grey) areas in a single model city (Leiden, The Netherlands), where we sampled the abundances of different mosquito life stages (eggs, larvae, adults) and the local microclimatic conditions. In this study, we focused on *Culex pipiens* s.l., which is the most common and abundant mosquito species in The Netherlands.

**Results:**

Our results show that while *Cx. pipiens* ovipositioning rates (number of egg rafts) and larval life stages were far more abundant in residential areas, adults were more abundant in parks. These results coincide with differences in the number of suitable larval habitats (higher in residential areas) and differences in microclimatic conditions (more amenable in parks).

**Conclusions:**

These findings suggest that *Cx. pipiens* dispersal may be considerably more important than previously thought, where adult *Cx. pipiens* seek out the most suitable habitat for survival and breeding success. Our findings can inform more targeted and efficient strategies to mitigate and reduce mosquito nuisance while urban green spaces are increased, which make cities more climate resilient.

**Graphical Abstract:**

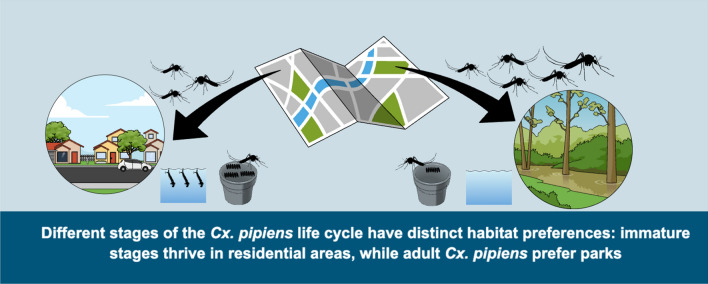

## Background

In recent decades, the world has witnessed a remarkable increase in urbanisation, and it is estimated that in 2050 > 70% of the world’s population will live in sprawling urban areas [1]. Urbanisation and urban areas have been associated with increased mosquito abundance due to the loss of natural predators and the availability of suitable breeding habitats [2, 3]. This increase in mosquito abundance, coupled with a higher density of human population, has increased the occurrence of mosquito-borne diseases in urban areas, with suburban residential areas and areas in proximity to parks being particularly vulnerable [4–6]. Moreover, the temporal occurrence of these outbreaks can be linked to extreme weather events, such as heatwaves, drought and heavy erratic rainfall, which are projected to increase due to global climate change and which are generally more extreme in cities [7–11]. As cities continue to expand and climate change impacts become more pronounced, there is an urgent need to address the challenges posed by increasing mosquito-borne disease occurrences [7, 12].

One of the key strategies employed by cities to become more climate resilient are nature-based solutions, such as establishing green spaces, promoting biodiversity and integrating nature into the urban fabric [13–15]. For instance, cities are constructing green infrastructure, such as wetlands and city parks, and retrofitting buildings with green roofs, to absorb and manage stormwater and mitigate the urban heat island effect. Currently, there is a worry that (some of these) climate adaptation strategies will affect mosquito-borne disease risk, but it is unclear whether these are detrimental or beneficial for mosquitoes [7, 13, 16]. While the need for such solutions is clear, the challenge lies in implementing them while minimizing the risk of mosquito-borne diseases and mosquito-related nuisances [7, 17]. This necessitates a comprehensive understanding of the ecological factors driving mosquito distribution in urban areas. While some general patterns regarding the effects of vegetation and the availability of container breeding sites are known, there are almost no empirical data on mosquito distribution within urban environments—comparing green to grey spaces—or what role green and grey spaces in cities might play in mosquito population dynamics [18–30]. For this, we need to understand the distribution of the different life stages of mosquitoes, which is essential for establishing links with environmental factors, such as microclimate.

To understand how the various mosquito life stages are distributed across the urban fabric, we set out to investigate how these life stages (eggs, larvae, adults) are distributed across contrasting urban environments (city parks vs. residential areas) and how this covaries with the local microclimatic conditions. To this end, we investigated these factors in the metropolitan area of Leiden (NUTS-3 region), a typical Northwestern European urban area with a highly heterogeneous urban landscape consisting of densely populated residential areas interspersed with parks. In this study, we focused on *Culex pipiens/torrentium* (hereafter: *Cx. pipiens*), which is the most common and abundant mosquito (Culicidae) species in The Netherlands and Northwestern Europe [31–33]. This species is particularly abundant in urban areas, utilising a variety of habitats ranging from artificial containers to natural stagnant waterbodies for breeding [34–37]. *Culex pipiens* is also the primary vector for the transmission of both Usutu virus (USUV) and West Nile virus (WNV) [38–41].

## Methods

### Study design

**Fig. 1:**
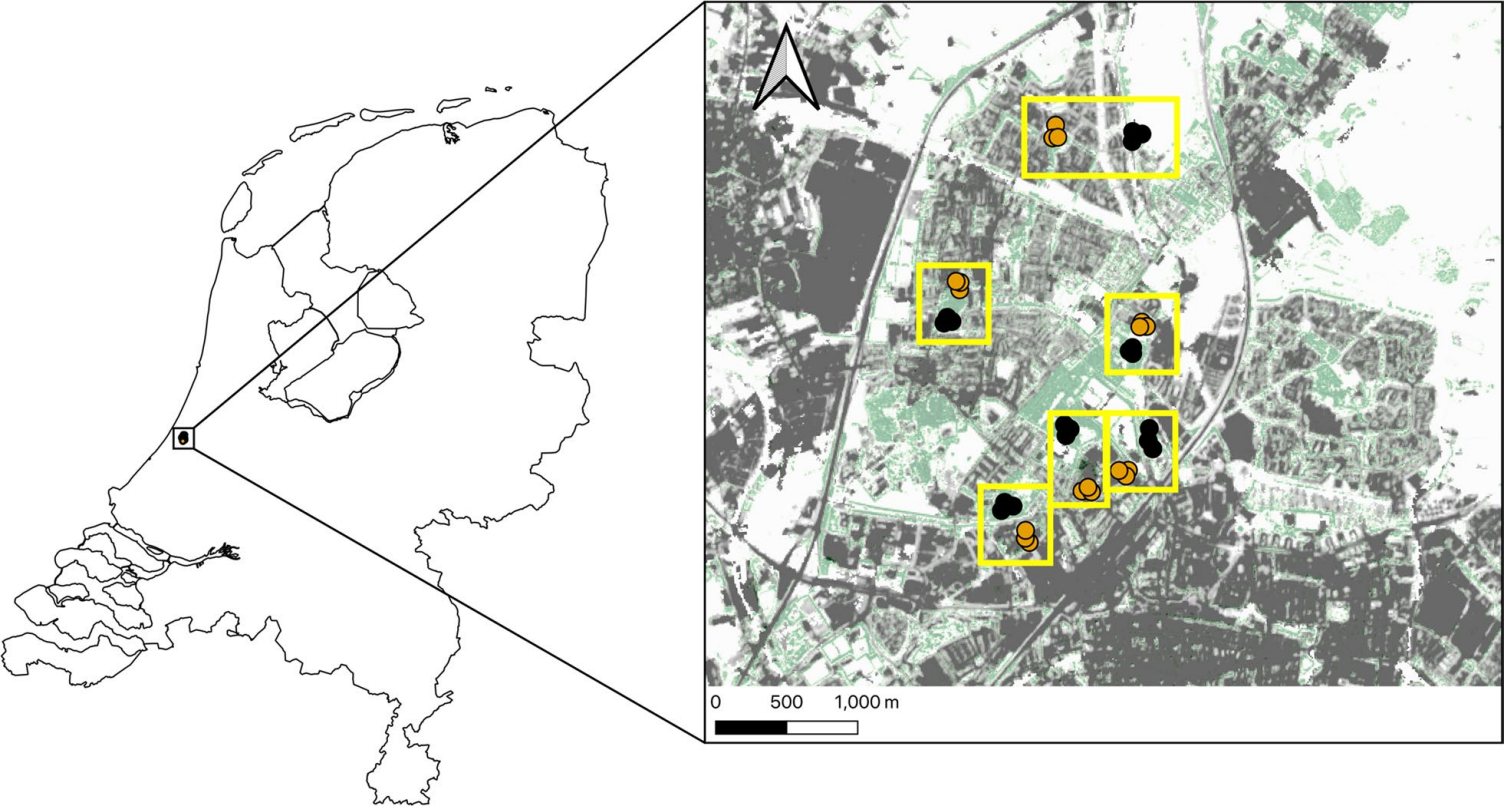
Map showing the locations of mosquito sampling in the city of Leiden, The Netherlands, in relation to the percentage tree cover (grey: artificial surfaces; green: tree cover). Black dots indicate park sites and orange dots indicate residential sites. Yellow boxes indicate the six paired locations. Maps created using QGIS (version 3.16, Hannover; Development Team, 2022) and Copernicus land cover data from 2018

A field study was conducted from 30 May to 8 July 2022, starting 2 weeks after a period of precipitation. This time frame enabled us to assess variations in the response of mosquito life stages to rainfall between residential areas and city parks, precipitation being one of the driving factors of mosquito abundances [42, 43]. We used a setup of six paired locations, each location pair consisted of three park sites (urban green areas) and three residential sites (urban grey areas) which were selected based on differences in human population density and vegetation composition (Fig. 1). As a proxy for residential areas, schoolyards in residential areas were chosen as they are relatively uniform in terms of vegetation and size and more accessible than privately owned spaces in residential areas.

### Sampling the abundances of adult *Cx. pipiens* mosquitoes

Adult mosquitoes were trapped every week during two subsequent trapping nights at all six paired locations (6 park and 6 residential). Per location three carbon dioxide-baited BG Pro traps (Biogents GmbH, Regensburg, Germany) were placed for a total of 36 trapping sites [44]. Traps were spaced approximately 30–40 m apart in a triangular pattern to prevent competition between traps caused by intersecting CO_2_ plumes [45]. We attached the traps to a tree or pole at a height of ~ 1.5 m, surrounded by high vegetation to provide shelter from the wind for the carbon dioxide plume [46, 47]. Carbon dioxide was generated using a modified sugar fermentation protocol by mixing 200 g granulated sugar, 5 g dried *Saccharomyces cerevisiae* yeast (Fermentis, SafSpirit FD-3), 5 g store-bought tomato paste and approximately 1.5 l tap water in 4-l jerrycan bags (Packforce, Jerrycan pouch) [47, 48]. The jerrycan bags were placed in the BG Pro bags and connected to the mosquito traps through silicone tubes (Ø7 mm; RubberBV, Hilversum, The Netherlands). Collected mosquitoes were separated by sex and the female mosquitoes were identified morphologically based on the characteristics outlined in Becker et al. [17]. *Culex pipiens* s.l. and its sibling species *Cx. torrentium* are challenging to differentiate reliably based on morphological characteristics of adult females and were therefore grouped together and referred to as *Cx. pipiens* [17, 35, 49].

### Measuring *Cx. pipiens* ovipositioning

*Culex pipiens* egg rafts were collected weekly during two subsequent trapping nights at all 36 sites within the 6 paired locations using three 5-l (202 × 171 mm) round black buckets (Dijkstra Plastics, Haaksbergen, The Netherlands), which were placed under each adult mosquito trap. To attract gravid mosquitoes and outcompete local breeding sites, we mimicked the nutrient level of a hypertrophic land puddle (100 mg/l N-total) [18, 50, 51]. To each bucket we added 4 l ditch water and dried cow manure (2.4% N; 1.5% P_2_O_5_; 3.1% K_2_O) to reach 100 mg/l N-total, which was then left to incubate for 1 week so that the microbial communities could stabilise [50]. To ensure that egg raft collection coincided with the adult mosquito trapping, a lid was placed on the bucket and removed during the two trapping nights. Thereafter, the egg rafts were counted and removed from the bucket to prevent any potential confounding effects of mosquito larval presence on oviposition behaviour [52].

### Counting *Cx. pipiens* breeding habitats and measuring occupancy

**Fig. 2:**
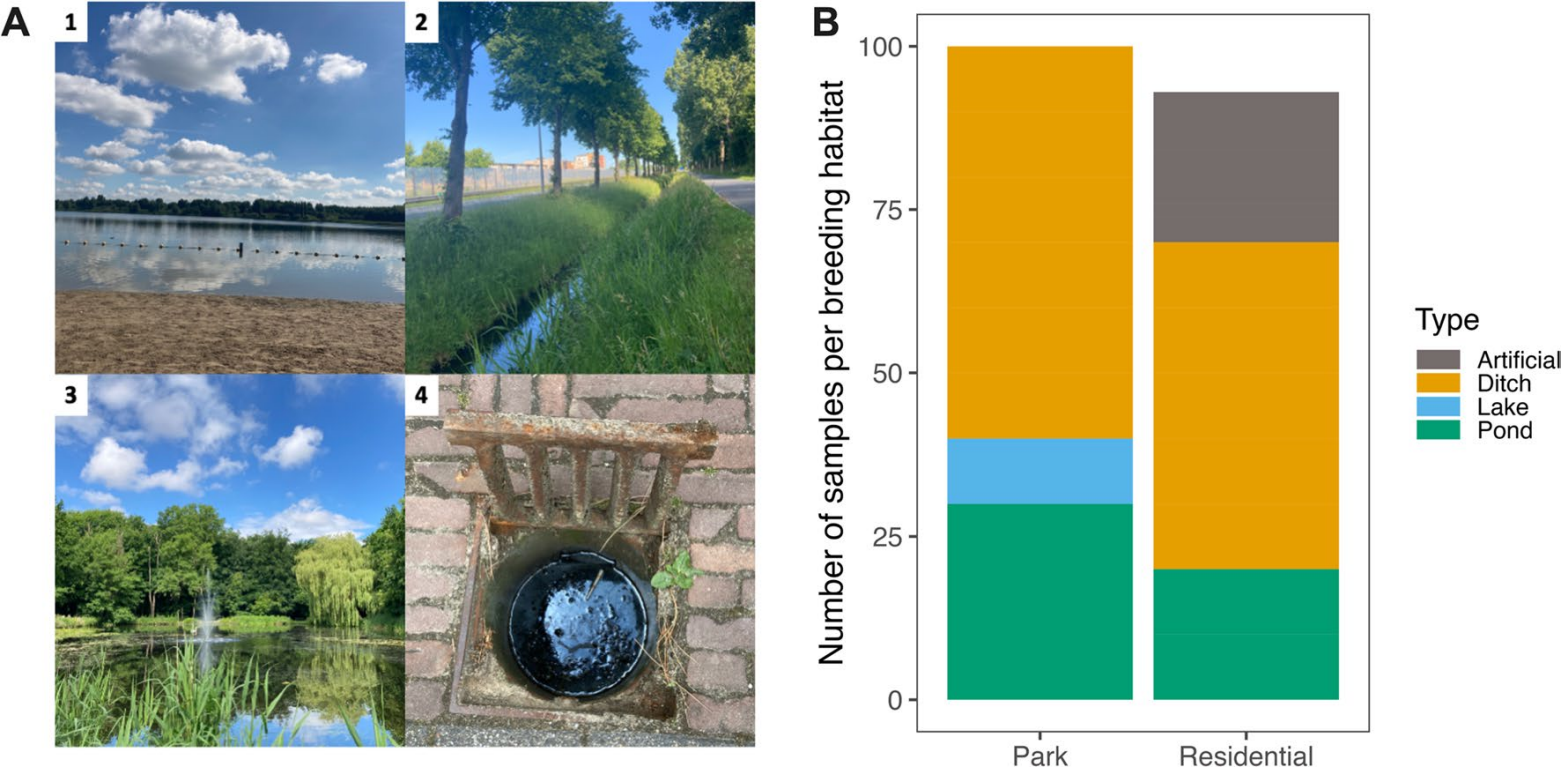
Examples of the four mosquito breeding habitat categories, lake (1), ditch (2), pond (3) and artificial (4) (**A**), and in relation to the number of samples per breeding habitat category in parks and residential areas (**B**)

*Culex pipiens* breeding habitats were counted and sampled for occupancy once in early June and 2 weeks after a period of precipitation. This method ensured that we only counted the breeding habitats that persisted long enough for adult *Cx. pipiens* to lay their eggs and for larvae to develop, which typically takes about 2 weeks under local climatic conditions [50]. For all 36 sites, we registered and sampled all potential breeding habitats within a 100-m radius using both larval dipping and eDNA collection methods. We utilised both approaches because they complement each other: larval dipping provides accurate estimates when mosquito numbers are high and habitats are small, while environmental DNA can detect mosquito presence in cases of low larval numbers and large habitats [53]. However, eDNA is also able to detect legacy mosquito presence via ‘zombie DNA’, e.g. eDNA is still present in the water or sediment after the adults emerged [54, 55]. This residual eDNA can persist for 1–4 days until it is fully degraded by microbial activity and abiotic factors such as UV-B and pH [56–59]. We defined four mosquito breeding habitat categories, lake, ditch, pond and artificial (Fig. 2), to examine the overall disparities in the number of mosquito breeding habitats between parks and residential areas. These mosquito breeding habitats were then sampled using a larval dipper (BioQuip, Compton, CA, USA) using the “simple scoop” dipping technique, during which larvae were counted [60]. Large habitats such as lakes, ditches and ponds were sampled ten times to get a representative sample, and confined artificial habitats, such as storm drains, were sampled once. For every habitat category encountered in the 100 m radius, a total of 1 l water was collected for eDNA analysis via subsampling with a pipet controller (Integra Bioscience) to improve detection probabilities [61].

### Extraction of eDNA from mosquito breeding habitats

Environmental DNA was collected using a 250-ml Sartorius filtering tower (Sartorius-stedim), a mobile vacuum pump (Datura Molecular Solutions) and multiple 0.45-micron polyethersulfone (PES) filters with a diameter of 47 mm (Tisch Scientific). To prevent cross-contamination, the Sartorius filtering tower was cleaned between filtrations with tap water (to remove large particles) and then soaked for 30 min in a 10% bleach solution to degrade remaining DNA. After filtration, the filter was immediately placed in a 2-ml centrifuge tube and completely immersed in 900 μl CTAB buffer. DNA extraction was performed using a modified PCI protocol followed by a column based DNA extraction as clean-up [62–67]. The PES filters were incubated at 65 °C for 10 min. Next, chloroform-isoamylalcohol (CI 24:1) was added to the filters and vortexed until completely disintegrated. The resulting mixture was centrifuged at 15,000 × g for 15 min, and 500 μl of the aqueous layer was transferred to a new tube. To this, 1 ml 96% ethanol and 180 µl 3 M NaOAc were added, followed by thorough vortexing. The tube was then placed in the freezer at −20 °C overnight for precipitation. For DNA purification, the tubes were centrifuged again, and the liquid was removed. The pellets were air-dried until no visible liquid remained and were then resuspended in 180 μl ATL buffer. DNA extraction was continued with the DNeasy Blood & Tissue kit (Qiagen) for DNA purification using the manufacture protocol. DNA was finally eluted in 200 μl AE buffer.

### Measuring *Cx. pipiens* eDNA traces in mosquito breeding habitats

We measured *Cx. pipiens* eDNA traces using *Cx. pipiens* s.l.-specific primers (forward: 5ʹ-CTGGAGCTTCAGTAGACT-3ʹ and reverse: 5ʹ-AGAGTAATTCCTGAAGATCG-3ʹ) in conjunction with a probe (5ʹ-[HEX]AGCAGGAATTTCATCATTTTAGGTGC[BHQ1]-3ʹ) targeting the COX1 genomic region. These primers and probe were designed and tested in silco using the Primer3 plug-in for Geneious, version R10 [68, 69]. For validation we used a mispriming library containing *Aedes albopictus*, *Culiseta annulata* and *Chironomus riparius*. The primers and probe were validated in vitro with a thermal gradient to select the optimal annealing temperature and test for cross amplification in the species from the mispriming library. *Culex pipiens* eDNA concentration was measured in triplicate using the Droplet Digital PCR system (ddPCR) with the Probe assay (Bio-Rad Laboratories, Inc.). The ddPCR reaction mix (22 μl) consisted of 11 μl of ddPCR^™^ Supermix for Probes, 1 μl of each primer (10 μM), 1 μl probe (5 μM), 7 μl RNase and DNase free-water, and 2 μl DNA template. To partition 20 μl of the PCR reaction mix into droplets, we used the QX200 Droplet Generator. PCR was performed in a Bio-Rad C1000 touch Thermal Cycler, using the following programme: 5 min at 95 °C, and 40 cycles of 30 s at 95 °C and 60 s at 52.3 °C with ramp rate of 2.0 °C/s, followed by 10 min at 98 °C. After amplification, the PCR plate was analysed using the QX200 Droplet Reader. We quantified the number of target copies using Bio-Rad's QuantaSoft software version 1.7.4. PCR replicates were consolidated into a single sample through QuantaSoft's merge function. The positive signal threshold was established based on a positive control sample, following QuantaSoft manual guidelines. We calculated the threshold values in test samples by determining the separation value between the threshold and the center of the negative droplet band, which was set at 1800 units above the average negative band. Droplets exceeding this threshold were counted as positive events. Blank samples containing only RNase and DNase free water were used as negative controls for the test samples. Sample count estimates were compared to the maximum confidence interval (95%) of the negative controls to ascertain whether DNA concentrations were statistically different from zero.

### Assessing microclimate

The microclimate at three parks and three residential areas sites were monitored continuously using a single open-source weather station for each site [70]. The weather stations were deployed on 30 May and retrieved on 8 July 2022 and recorded the following variables at a 5-min interval: air temperature (°C; accuracy ± 1 °C), relative humidity (%; accuracy ± 3%) and wind speed (m/s; accuracy ± 0.1 m/s). These climatic variables are known to be of key importance for mosquito survival, development and behaviour [42, 43, 71, 72].

### Statistical analysis

We tested overall differences in adult *Cx. pipiens* abundances, egg rafts, larvae counts and eDNA traces between parks and residential areas over time using generalized linear mixed models (GLMMs). We used weekly trap data, observed egg rafts, larvae counts and eDNA concentration as response variables. For these models we used site (parks vs. residential areas) and week as predictor variables, with paired locations as random effects. We employed a Poisson distribution with a log-link function and assessed predictors using an analysis of variance (ANOVA). To test for differences in microclimatic conditions between parks and residential areas, we first calculated the weekly minimum, mean and maximum values for temperature, relative humidity and wind speed. Minimum wind speed was always zero and was excluded from further analysis. A linear mixed-effect model (LMEM) was run to test for differences between parks and residential areas over time for each of the three variables, temperature, humidity and wind speed, and three levels, minimum, mean and maximum. A Bartlett test was conducted to assess the homogeneity of variances between parks and residential areas for each of the three microclimatic variables. All data analysis was conducted with RStudio (R version 4.1.0; R Core Team, 2021) [73]. All relevant assumptions were checked before the statistical tests were carried out.

## Results

### Differences in adult *Cx. pipiens* abundances between parks and residential areas

**Fig. 3:**
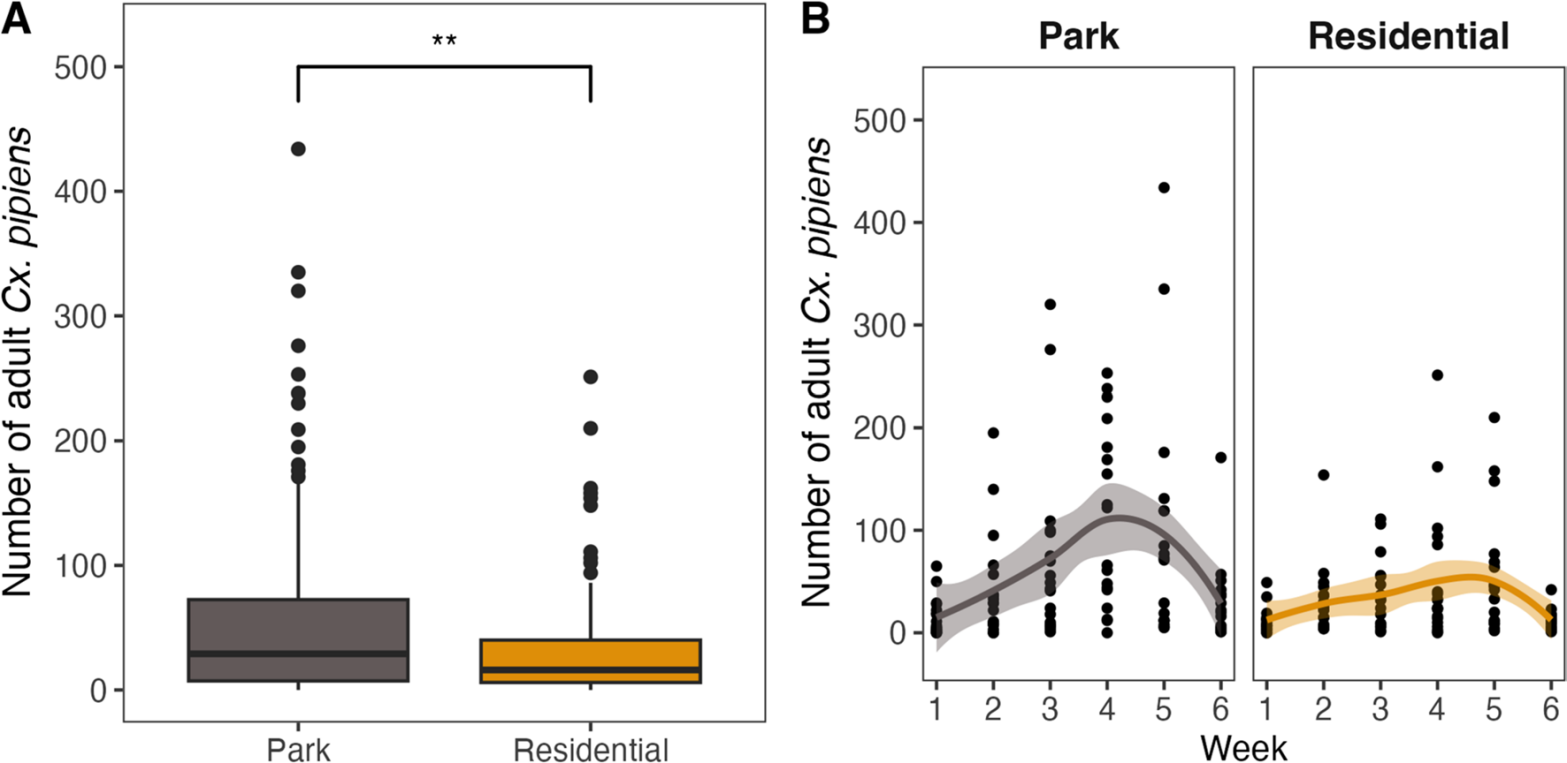
Total number of female *Culex pipiens* mosquitoes trapped per week per trap at each of the park and residential areas (**A**) and total numbers separated by week (**B**); line is mean trend with a 95% confidence interval

We captured a total of 10,227 adult female *Cx. pipiens* mosquitoes. In parks we observed almost double the number of mosquitoes (6624) compared to (3603) residential areas. We fitted a GLMM to predict the number of adult *Cx. pipiens*, with an R^2^ of 0.98, of which 0.49 is related to the fixed effects (e.g. park vs. residential area and week). When we look at the weekly number of adult *Cx. pipiens* per trap, we observe that this difference is overall statistically significant (Wald test, *χ*^2^ = 6.7523, df = 1, *Ρ* < 0.01; Fig. 3A), as is the interaction with week (Wald test, *χ*^2^ = 82.6743, df = 5, *Ρ* < 0.001; Fig. 3B).

### Differences in *Cx. pipiens* oviposition between parks and residential areas

**Fig. 4:**
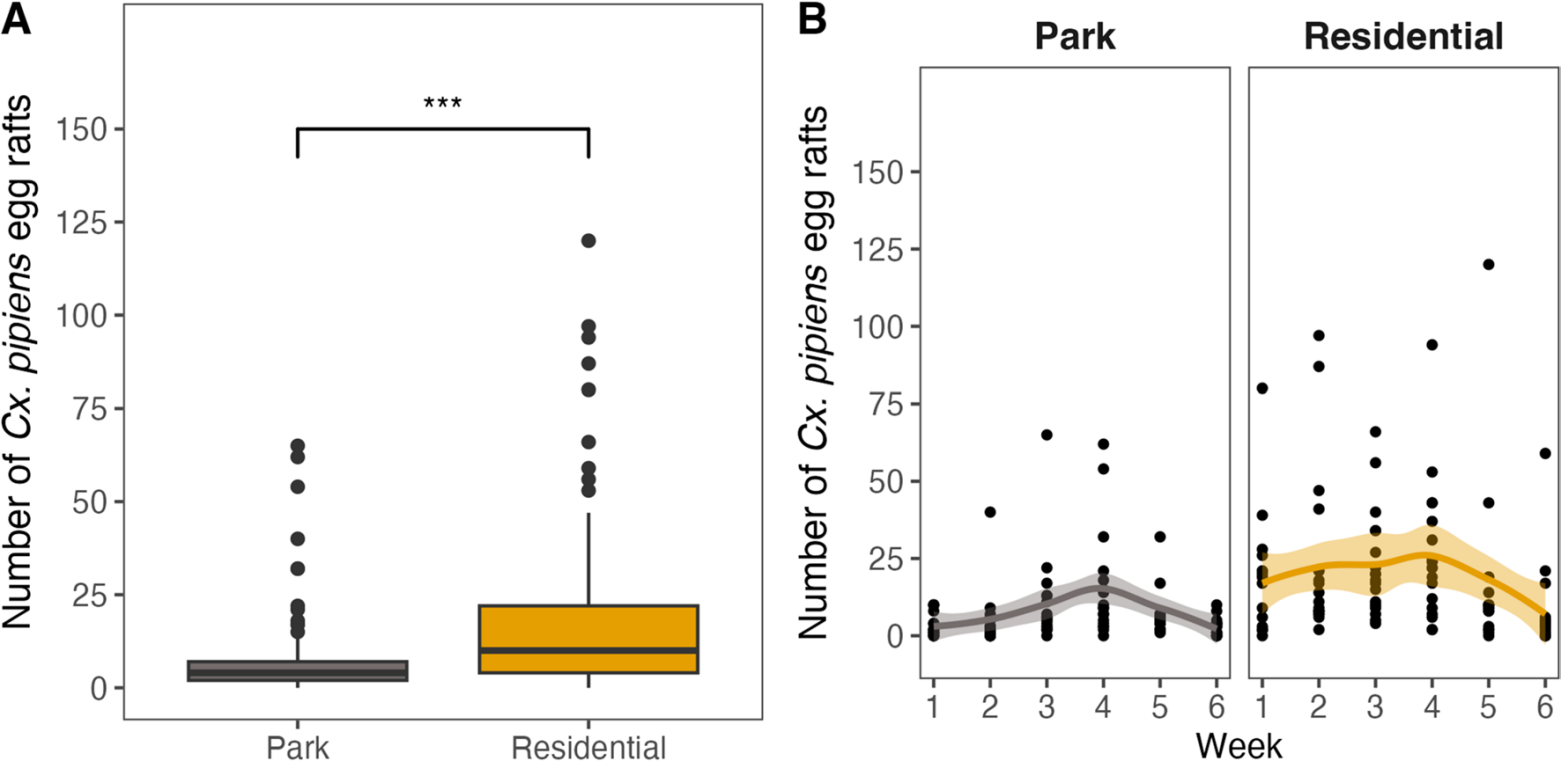
Total number of *Culex pipiens* egg rafts counted per week per ovipositioning bucket at each of the park and residential sites (**A**) and total numbers separated by week (**B**); line is mean trend with a 95% confidence interval

We counted a total of 2841 *Cx. pipiens* egg rafts. In residential areas we observed almost three times more egg rafts (2067) compared to parks (774). We fitted a GLMM to predict the number of *Cx. pipiens* egg rafts, with an *R*^2^ of 0.88, of which 0.73 is related to the fixed effects. When we look at the weekly number of *Cx. pipiens* egg rafts per ovipositioning bucket, we observe that this difference is overall statistically significant (Wald test, *χ*^2^ = 39.233, df = 1, *Ρ* < 0.001; Fig. 4A), as is the interaction with week (Wald test, *χ*^2^ = 86.810, df = 5, *Ρ* < 0.001; Fig. 4B).

### Differences in number of *Cx. pipiens* larvae between parks and residential areas

**Fig. 5:**
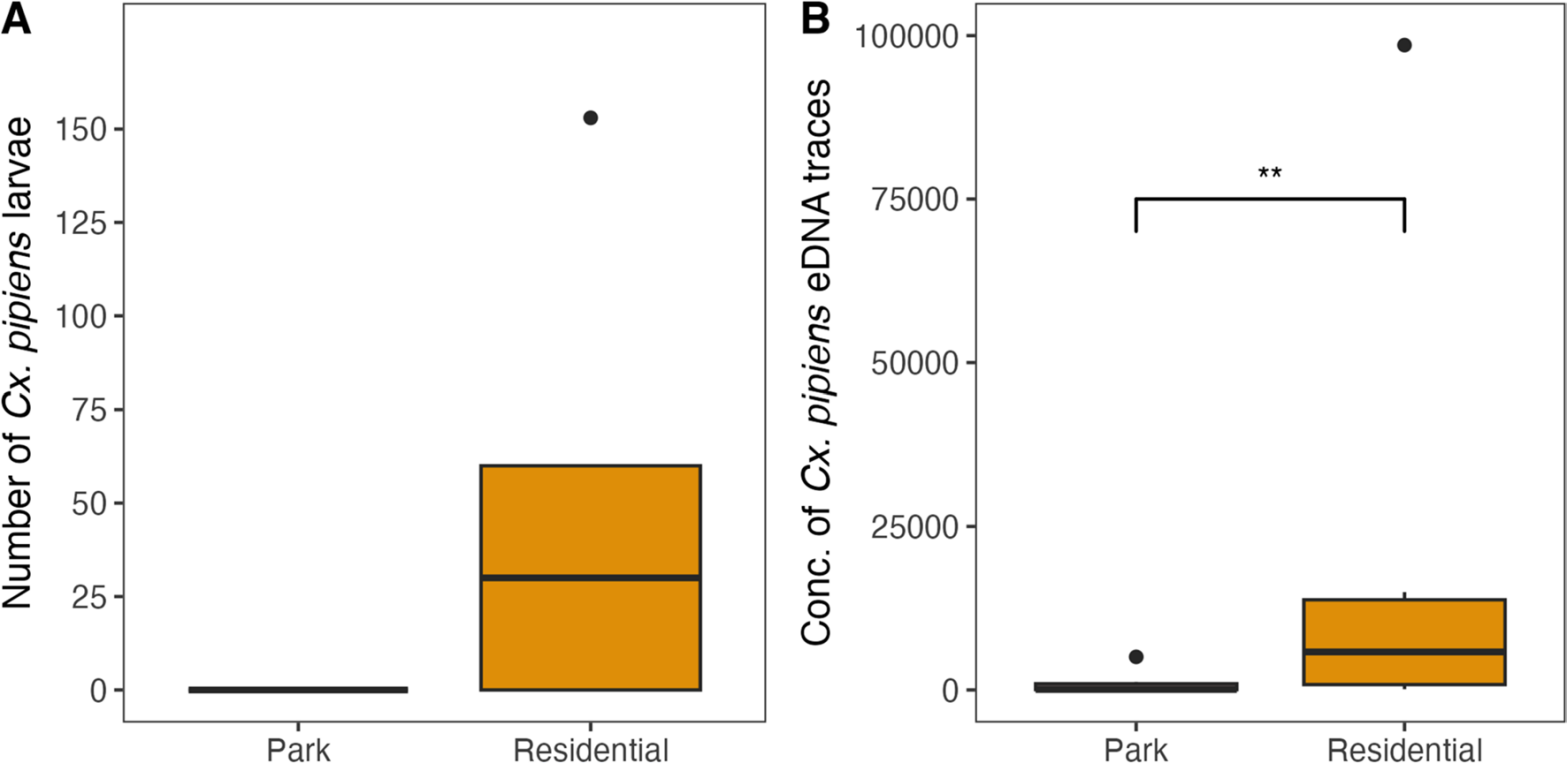
The total number of *Cx. pipiens* larvae counted (**A**) and eDNA traces measured (**B**) per sampled breeding habitat at each park and residential site

We counted a total of 665 *Cx. pipiens* larvae, all observed in storm drains in residential areas. These observations were confirmed by the outcome of our eDNA measurements, which detected on average 20 times higher concentrations of *Cx. pipiens* eDNA traces in breeding habitats in residential areas compared to breeding areas in parks. We fitted a GLMM to predict the concentrations of *Cx. pipiens* eDNA traces, with an *R*^2^ of 0.80 of which 0.38 is related to the fixed effect of parks versus residential. The difference in eDNA concentrations is overall statistically significant (Wald test, *χ*^2^ = 9.0782, df = 1, *Ρ* < 0.01; Fig. 5B).

### The microclimate in parks and residential areas showed remarkable similarity in maximum temperature, minimum relative humidity, and mean and maximum wind speed

**Fig. 6:**
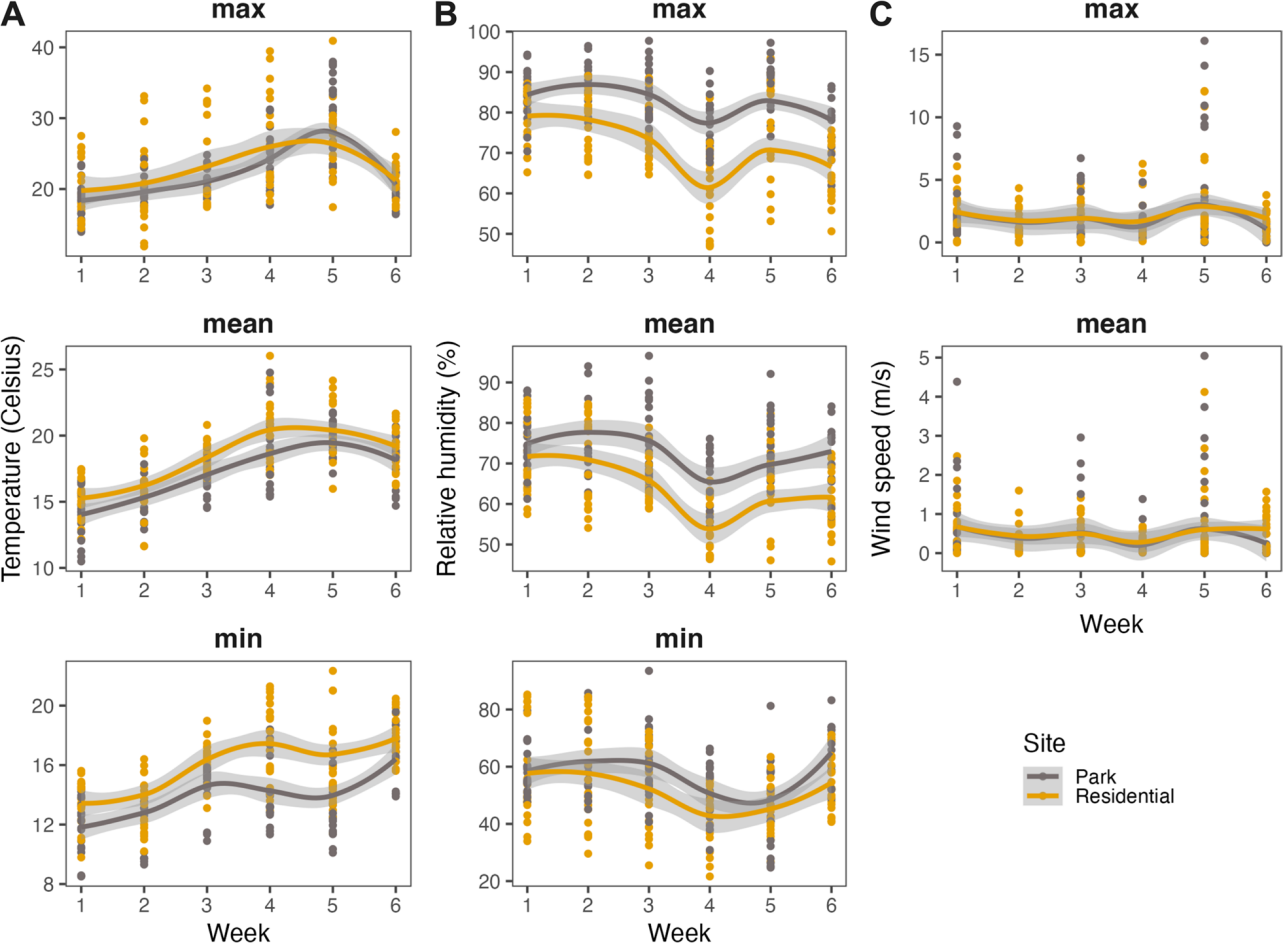
Microclimatic conditions. Temperature (**A**), relative humidity (**B**) and wind speed (**C**) at parks and residential areas, summarised per week and weather station

The microclimate in parks and residential areas showed remarkable similarity in temperature (maximum), relative humidity (minimum) and wind speed (mean, maximum). Maximum temperature in parks was indistinguishable from that in residential areas (22.9 vs. 22.9 °C) (LMER, *t* = −0.0232, df = 4, *Ρ* = 0.983; Fig. 6A). In contrast, mean temperatures were 1.1 °C lower in parks compared to residential areas (17.2 vs. 18.3 °C) (LMER, *t* = −3.04, df = 4, *Ρ* < 0.05; Fig. 6A), and minimum temperatures in parks were 2.1 °C lower (13.7 vs. 15.8 °C) (LMER, *t* = −2.68, df = 4, *Ρ* = 0.0551; Fig. 6A). For humidity we found that parks maintained 10.4% higher levels of maximum relative humidity (82.9% vs. 72.5%) (LMER, *t* = 3.72, df = 4, *Ρ* < 0.05; Fig. 6B), 7.7% higher levels of mean relative humidity (72.4% vs. 64.7%) (LMER, *t* = 3.22, df = 4, *Ρ* < 0.05; Fig. 6B) and 3.4% higher levels of minimum relative humidity (55.3% vs. 51.9%) but this was not statistically significant (LMER, *t* = 0.447, df = 4, *Ρ* = 0.678; Fig. 6B). Only small differences were observed for wind speed. The maximum wind speed was slightly higher in parks compared to residential areas (2.4 m/s vs. 2.2 m/s), but this was not statistically significant (LMER, *t* = 0.165, df = 4, *Ρ* = 0.878; Fig. 6C). Mean wind speed showed a similar pattern: small non-significant differences between residential areas and parks (0.59 m/s vs. 0.52 m/s) (LMER, *t* = 0.131, df = 4, *Ρ* = 0.902; Fig. 6C). Interestingly, when examining the variance between parks and residential areas, wind speed demonstrates significantly higher variability in parks (3.82 vs. 2.46 (m/s)^2^) compared to residential areas (Bartlett test, *K*^2^ = 17.188, df = 1, *Ρ* < 0.0001). Conversely, for both temperature (Bartlett test, *K*^2^ = 0.96025, df = 1, *Ρ* = 0.327) and relative humidity (Bartlett test, *K*^2^ = 0.059317, df = 1, *Ρ* = 0.808), no significant differences in variance were observed between parks and residential areas.

## Discussion

Our results indicate that while adult *Cx. pipiens* mosquitoes were more abundant in park sites, *Cx. pipiens* ovipositioning (eggs) and larval stages were far more abundant in densely populated residential areas. These results coincide with differences in the availability of artificial breeding habitats and microclimatic conditions. These results suggest that, depending on the life stage, different habitat characteristics are governing *Cx. pipiens* distributions. It is, however, important to realize that all results were derived from a single metropolitan area in Northwestern Europe and for a single species complex. Although Leiden is a typical European metropolitan area with residential areas interspersed with green parks, it remains to be tested whether patterns observed here reflect those occurring in other regions or climate zones and for different species. Given the fact that most cities are composed of separated grey and green spaces, our results might hold for other urban environments where *Cx. pipiens* is the most abundant species.

### Parks are more populated by adult *Cx. pipiens* mosquitoes

Vegetation structure plays an important role in modifying microclimatic conditions such as temperature, humidity and wind speed by acting as a buffer, which are all linked to adult mosquito survival and development [42, 71, 72, 74]. Conversely, built-up structures, such as roads and buildings, also modify these microclimatic conditions, but then in the opposite direction, resulting in the urban heat island effect [75]. Indeed, our results show that parks were cooler and maintained higher relative humidity levels compared to residential areas, which is line with literature and promotes mosquito abundance based on their temperature preferences [74–77]. Adult mosquitoes are fragile insects that dry out quickly and are easily overwhelmed by strong gusts of wind [37, 78]. Wind may also impact host-seeking behaviour, which depends on mosquito olfactory senses, that require particles moving past them, making it necessary for mosquitoes to sense these compounds downwind [79]. For mosquitoes to navigate towards these sources, the prevailing wind speed must be lower than their average flight speed of 1 m/s [78]. Our results show that there is no difference in overall wind speed between parks and residential areas. However, we did measure higher variance in wind speed within parks. These results suggest that wind speed provides no proximate explanation to differences in adult *Cx. pipiens* abundance between the grey and green urban habitats. Overall, our results suggest that the distribution of adult *Cx. pipiens* mosquitoes is likely impacted by the observed differences in humidity and temperature between residential and green areas. However, we did not confirm the relation between microclimate and adult *Cx. pipiens* mosquito abundances because of restraints in our experimental design.

### Residential areas are more populated by immature *Cx. pipiens* life stages

Residential areas generally host an abundance of artificial substrates (containers, floodwater drains, etcetera) in which temporal water bodies can form after precipitation [2, 21, 22]. In parks, characterised by permeable soils and permanent water bodies, precipitation will either infiltrate into the soil or flow towards permanent water bodies before immature mosquitoes can emerge as adults. These permanent water bodies often contain an abundance of mosquito predators such as fish, amphibians and dragonfly larvae [72–75]. Even though we only sampled publicly accessible mosquito breeding habitats in the residential environment and all breeding habitats in the parks, we only visually observed larvae in residential areas in storm drains, which are a well-known habitat for *Cx. pipiens* [80, 81]. These habitats were often littered with organic material and devoid of any potential mosquito predator. Interestingly, with eDNA we were also able to detect *Cx. pipiens* eDNA traces in almost all other sampled potential breeding habitats, albeit in far lower concentrations than in the storm drains where we also found larvae. This suggests that *Cx. pipiens* mosquitoes do oviposit in all available breeding habitats, but that the larvae do not reach high enough abundances to be detected with larval dipping techniques [60, 82]. This could be due to low ovipositioning rates because more attractive habitats were available and/or that mosquito larvae were eaten by predators before they could reach detectable densities [18, 83–87]. Nevertheless, these permanent water bodies do have a large surface area where immature mosquito larvae can occur, particularly between water bank vegetation and within small, shallow, semi-connected water bodies along the shoreline [18, 87–89]. It is plausible that these habitats may function as reservoirs, maintaining low densities of *Cx. pipiens* mosquitoes. These *Cx. pipiens* mosquitoes might subsequently migrate to more suitable temporary water bodies following a precipitation event [85, 86]. In summary, these findings show higher larval *Cx. pipiens* abundances, higher *Cx. pipiens* ovipositioning rates and higher availability of suitable breeding habitats in residential areas, implying a series of important pull factors for gravid *Cx. pipiens* mosquitoes looking for a suitable site for egg deposition.

### Short-scale migration of *Cx. pipiens* in the urban environment?

Mosquitoes require an aquatic habitat to oviposit and for larvae to feed and develop via four successive molts into pupae from which adults can emerge [17, 18, 37]. These adult mosquitoes fly out and disperse to seek shelter against the elements, nectar to feed, a bloodmeal to develop eggs and suitable breeding habitats to oviposit their eggs [71, 78]. We observed that parks likely provide a more favourable environment for adult *Cx. pipiens* with their favourable microclimates. We also observed that residential areas provide a more suitable environment for the immature life stages of *Cx. pipiens*. As an explanation for this remarkable division, we propose that short distance *Cx. pipiens* mosquito migration between the various urban habitats is underlying this pattern. According to this hypothesis, the bulk of the urban *Cx. pipiens* emerges from artificial breeding habitats in residential areas and a fair number of them will move towards parks to rest, mate and seek a blood meal. As parks lack the required predator-free habitat for egg deposition, they (unintentionally) migrate back to residential areas to deposit their eggs. While we cannot exclude the possibility that the urban environment has two distinct populations that do not mix, this is unlikely given the previously reported dispersal distances of *Cx. pipiens* [71, 90].

### Implications for urbanising landscapes under a changing climate

Nature-based solutions play a crucial role in enhancing the climate resilience of cities, with a significant focus on increasing biodiversity and integrating nature into the urban fabric [13–15]. Although our study was not designed to assess the effects of nature-based solutions, our results do offer valuable insights into how these solutions could potentially influence *Cx. pipiens* populations. Our results suggest that increasing green areas in the form of high woody vegetation in residential areas to reduce the urban heat island effect will not necessarily increase the total number of adult *Cx. pipiens*, although there are competing factors at play. Based on our results we cannot make any inference on how many *Cx. pipiens* that emerge from residential breeding habitats die on their way to find sheltered environments. Having more sheltered (nature-based) environments in residential areas could potentially increase adult survival, thus increasing total abundance in the urban environment. On the other hand, greening the city could potentially reduce the possible number of (artificial) temporal breeding habitats by increasing permeable surfaces like grasslands and green roofs, creating new permanent water bodies can help absorb rainwater, which may result in a reduction in stormwater runoffs. The latter set of measures will likely reduce the number of *Cx. pipiens* larvae via the reduction of (potential) artificial breeding habitats and increase predation in newly formed permanent water bodies that are linked to well-established ecosystems [91–94]. Our study shows that while *Cx. pipiens* mosquitoes do oviposit in permanent water bodies, we did not detect any *Cx. pipiens* larvae. This might be due to the presence of mosquito predators [18, 83–85]. This would imply that new permanent water bodies should be linked to existing water bodies with a well-established ecosystem, from which predator species can colonise these new habitats [18, 95].

## Conclusions

Our study suggests that, based on the results from a single metropolitan area in Northwestern Europe, *Cx. pipiens* mosquitoes spend their adult life mostly in parks, where a variety of hosts (mainly bird and mammal species) co-occur and more suitable microclimates can be found (more humid and more buffered temperatures). In sharp contrast, we show that immature *Cx. pipiens* life stages are almost exclusively spent in densely populated residential areas, with their abundance of artificial habitats (temporal with eutrophic conditions). Overall, these results highlight the importance of urban landscape structure to understand mosquito distributions and suggest that *Cx. pipiens* dispersal may be considerably more important than previously thought, where adult *Cx. pipiens* mosquitoes seek out the most suitable habitat for survival and ovipositioning. Moreover, our findings suggest that enhancing the climate resilience of urban areas through nature-based solutions, while considering and mitigating the potential risks associated with their implementation, will not necessarily increase the population of adult *Cx. pipiens* mosquitoes.

## Data Availability

All data are available upon request to the corresponding author.

## Abbreviations

ANOVA: Analysis of variance
BHQ1: Black Hole Quencher 1
CI: Chloroform-isoamyl alcohol
COX1: Cytochrome c Oxidase Subunit 1
CTAB: Cetyltrimethylammonium bromide
ddPCR: Digital Droplet Polymerase Chain Reaction
df: Degrees of freedom
DNA: Deoxyribonucleic acid
DNase: Deoxyribonuclease
eDNA: Environmental DNA
G: Gravitational force
GLMM: Generalized Linear Mixed Model
HEX: 6-carboxyhexachlorofluorescein
LMEM: Linear mixed-effect model
NUTS-3: Nomenclature of Territorial Units for Statistics - Level 3
PCR: Polymerase Chain Reaction
PCI: Phenol-chloroform-isoamyl alcohol
PES: Polyethersulfone
pH: Potential of hydrogen
RNase: Ribonuclease
s.l.: Senso lato
USUV: Usutu virus
WNV: West Nile virus
